# Use of IFN-Based Biotherapeutics to Harness the Host Against Foot-And-Mouth Disease

**DOI:** 10.3389/fvets.2020.00465

**Published:** 2020-08-11

**Authors:** Gisselle N. Medina, Teresa de los Santos, Fayna Diaz-San Segundo

**Affiliations:** ^1^Plum Island Animal Disease Center (PIADC), ARS, USDA, Orient Point, NY, United States; ^2^Kansas State University, College of Veterinary Medicine, Manhattan, KS, United States

**Keywords:** foot-and-mouth disease virus (FMDV), interferon (IFN), antivirals, biotherapeutics, IFN-α, IFN-γ, IFN-λ, IFN-ω

## Abstract

Foot-and-mouth disease (FMD) is a highly contagious vesicular disease of cloven-hoofed animals that severely constrains international trade of livestock and animal products. Currently, disease control measures include broad surveillance, enforcement of sanitary policy, and use of an inactivated vaccine. While use of these measures has contributed to eliminating foot-and-mouth disease virus (FMDV) from a vast area of the world, the disease remains endemic in three continents, and outbreaks occasionally appear in previously declared FMD-free zones, causing economic and social devastation. Among others, a very fast rate of viral replication and the need for 7 days to achieve vaccine-induced protection are the main limitations in controlling the disease. New fast-acting antiviral strategies targeted to boost the innate immunity of the host to block viral replication are needed. Here we review the knowledge on the multiple strategies FMDV has evolved to block the host innate immunity, with particularly focus on the past and current research toward the development of interferon (IFN)-based biotherapeutics in relevant livestock species.

## Introduction

### The Disease: Foot-And-Mouth Disease

Foot-and-mouth disease (FMD) is one the most serious livestock diseases that affects cloven-hoofed animals including cattle, swine, sheep, and goats as well as numerous species of wild species (1). The disease displays high morbidity but is usually not lethal, except when it affects young animals that may develop myocarditis. Infected animals secrete copious amounts of virus particles before the onset of the clinical phase of the disease. Typical FMD clinical signs include fever and the appearance of vesicular lesions on the tongue, mouth, feet, and teats. Among ruminants that recovered from the disease, a relatively large number become asymptomatic virus carriers (2, 3), although it is not clear what is the contribution of these carrier animals to disease transmission in nature (4). The World Organization for Animal Health (OIE) lists FMD as a reportable disease and therefore, by law, participating nations are required to inform the organization about all FMD outbreaks. OIE member nations with reported cases of FMD are forbidden to engage in trading of FMD-susceptible animals or their products. Thus, the presence of FMD in a country can have severe economic consequences.

Different interventions to control an FMD outbreak include restriction of susceptible animal movement, slaughter of infected/contact animals, decontamination of infected and surrounding premises, and vaccination. Vaccination is an option used mostly in countries in which FMD is endemic, but disease-free nations prefer to abstain from such practice. In general, FMD-free countries that occasionally opted to vaccinate to better contain the outbreak did slaughter all vaccinated animals to regain commerce rights faster as occurred in the 2001 outbreak in the UK and the Netherlands (5, 6). The current approved FMD vaccine consists of purified chemically inactivated virus [binary ethylenimine (BEI)-treated] formulated with oil-based or aluminum adjuvants that induces serotype-specific protection in approximately 7 days, and it is applied with a boosting protocol for ensuring long-term protection (7). While this vaccine has been successfully used for many decades leading to disease eradication of a vast area of our planet, challenges remain. FMD is endemic in most of Africa and Asia, and occasionally epizootics appear in South America or in nations that have been disease-free for many years, as it happened in the UK, the Netherlands, South Korea, Taiwan, and Japan (8). Novel vaccine technologies have been developed, but to this end, none of them has fully addressed the limitations of the commercially available vaccine or is currently approved for massive use (9, 10). Alternatives or additional therapeutics that could complement, or in some instances substitute for vaccination protocols, include the use of antivirals and biotherapeutics that act quickly prior to induction of vaccine-induced immunity. The development of such molecules requires a thorough understanding of the biology of the virus and its intricate interactions particularly, with the innate immune molecular and cellular mechanisms evolved by the host.

### The Agent

Foot-and-mouth disease virus (FMDV) is a member of the *Aphthovirus* genus within the *Picornaviridae* family, and it is the etiologic agent of FMD (1). The virus contains a single-stranded RNA of positive polarity. Its genome of ~8,500 nucleotides consists of a long open reading frame (ORF), flanked by a 5′ and a 3′-untranslated region (-UTR). The ORF encodes a polyprotein of about 2,300 amino acids which is processed by virus-encoded proteases. Processing results in the generation of precursors and mature protein products including: four structural [1A (VP4), 1B (VP2), 1C (VP3), 1D (VP1)] and ten non-structural (NS) proteins [L^pro^, 2A, 2B, 2C, 3A, three distinct copies of 3B (VPg), 3C^pro^, and 3D^pol^]. Due to high genetic variability, FMDV is categorized in seven distinct serotypes, A, Asia-1, C, O, and Southern African Territories 1–3 (SAT 1–3), and numerous subtypes or topotypes. Upon infection, the virus spreads very rapidly usually achieving 100% morbidity. Depending on the route of entry, less than 10 tissue culture infectious doses are required to infect and cause disease in animals (11). In fact, FMDV is one of the fastest replicating RNA viruses in nature, taking as little as 3–4 h to induce cytopathic effects in susceptible tissue culture cells. One could envisage that during FMDV replication, almost every component of the virus must play a role in dampening interfering cellular responses to allow such rapid virus replication.

### Innate Immunity and Interferon Activation

**Figure 1:**
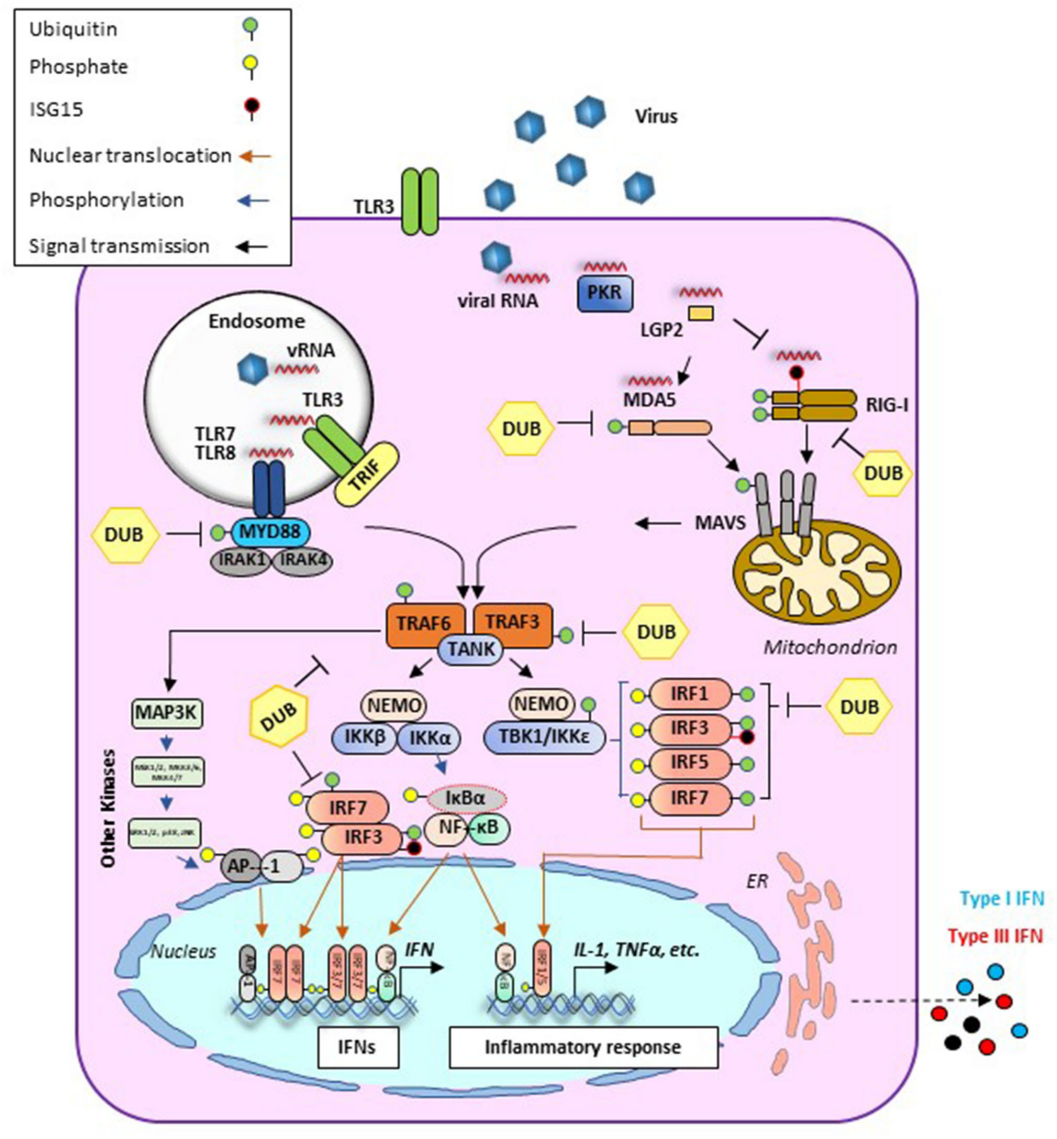
Antiviral signaling pathways induced during viral infection. Cellular detection of microbial molecules known as pathogen-associated molecular patterns (PAMPs, i.e., viral RNA) is mediated by pattern recognition receptors (PRRs) including cytosolic RNA sensors (i.e., RIG-I, MDA-5, or LGP2) and/or membrane-bound TLRs. PAMP/PRR interaction activates signal transduction cascades (*black arrows*) that result in the production of IFN and inflammatory cytokines. RIG-I and MDA5 contain two caspase recruitment domains (CARD) and an RNA helicase domain. In the case of RIG-I, ubiquitination (*green circles*) is required for its effective activation. Activated signals from either RIG-I or MDA5 are transmitted downstream via the mitochondrial adaptor MAVS resulting in the formation of MAVS filaments. At this stage, different PTMs such as ubiquitination or ISGylation (*black circles*) can regulate their functions. Endosomal RNAs are detected by TLR3 or TLR7/8 which signal through adaptor proteins TRIF and MyD88, respectively. MyD88 uses other adaptors, IRAK1/4, to allow for interaction with TRAF proteins. In addition to their role as adaptor proteins, TRAFs also serve as E3 ubiquitin (Ub) ligases to regulate signaling. TRAF-mediated induction of poly-Ub is sensed by NEMO, thus recruiting downstream effector kinases such as TBK1 or IKK. These proteins form different signaling complexes (i.e., NEMO/TBK1 and NEMO/IKK), leading to phosphorylation (*blue arrows*) of transcription factors IRF3/7 (to a lesser extent IRF1 and IRF5 are also phosphorylated). IRF phosphorylation triggers dimerization and translocation (*orange arrows*) to the nucleus where they bind mainly to IFN promoters/enhancers. Alongside with this pathway, TRAF6-E3 ligases can activate MAPK3 and other kinases including ERK1/2 and JNK which phosphorylate the components of the AP1 heterodimer, allowing for translocation to the nucleus and binding to the IFNβ promoter/enhancer to activate transcription. Activated IKK also phosphorylates IκB, releasing NF-κB, which then translocates to the nucleus and binds at the IFNβ promoter. AP-1, activating protein 1; CARD, caspase activation and recruitment domain; DUB, deubiquitinase; ER, endoplasmic reticulum; IκB, inhibitor of KB kinases; IKK, IκB kinase; IL, interleukin; IRAK, interleukin-1 receptor-associated kinase; IRF, IFN regulatory factor; LGP2, laboratory of genetics protein 2; MAPK, mitogen-activated protein kinase; MAVS, mitochondrial antiviral signaling protein; MDA5, melanoma differentiation-associated gene; MyD88, myeloid differentiation primary response protein 88d; NEMO, NF-κB essential modulator; NF-κB, nuclear factor-κB; PKR, protein kinase R; PTM, posttranslational modification; RIG-I, retinoic acid-inducible gene I; TANK, TRAF family member-associated NF-κB activator 1; TBK, TANK binding kinase; TLR, Toll-like receptor; TRAF, TNF receptor associated factor; TRIF, TIR-domain-containing adapter-inducing interferon-β.

**Figure 2:**
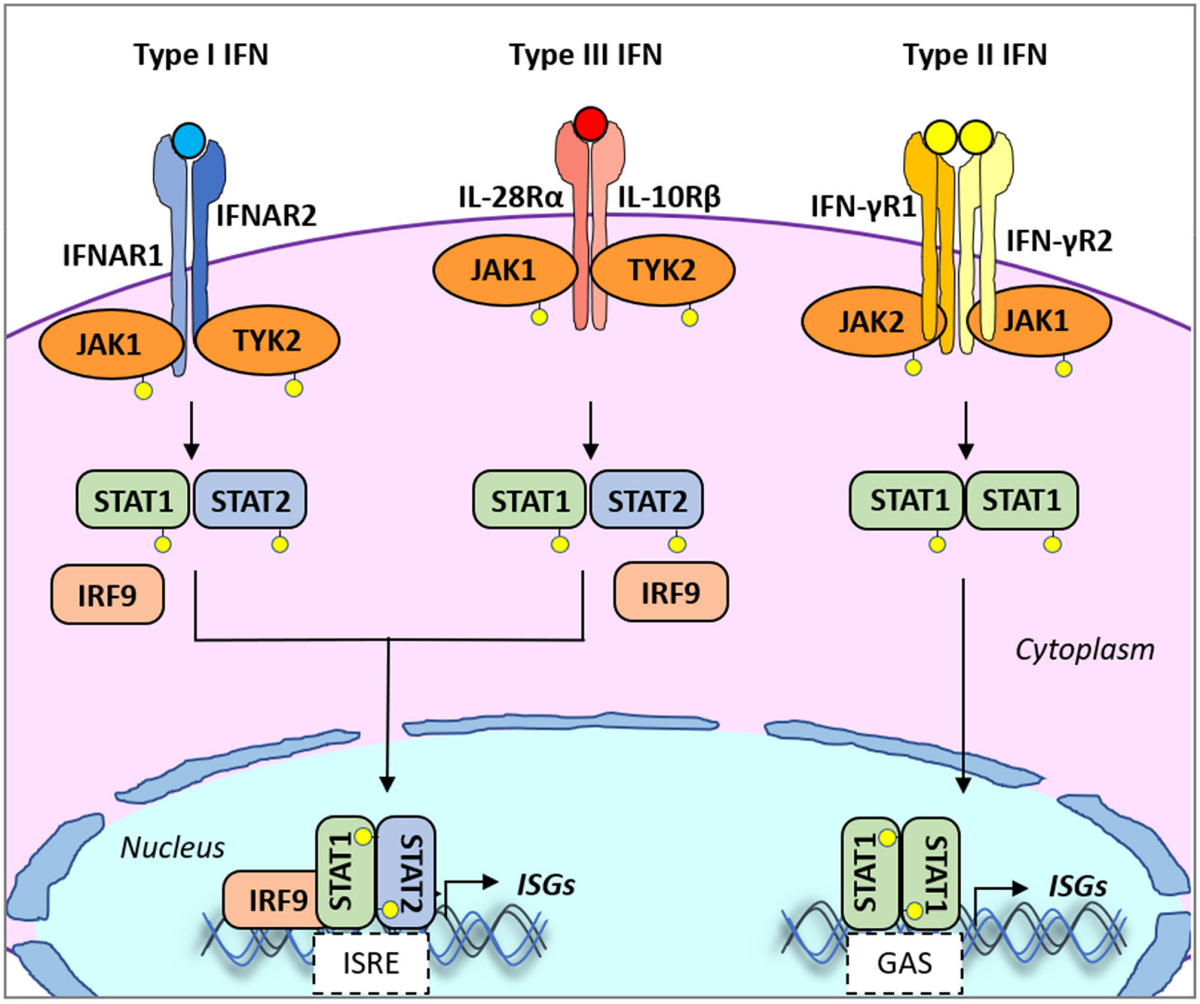
Type I, II, and III interferon (IFN)-mediated signaling. All type I and type III IFN subtypes bind to respective receptors, IFNAR1/IFNAR2 and IFNLR1/IL10R2. These interactions trigger the phosphorylation of JAK1 and TYK2 kinases which in turn phosphorylate STAT1 and STAT2. JAK2 mediates type III IFN-dependent STAT phosphorylation. Phosphorylated heterodimers of STAT1/STAT2 bind to IRF9, forming the ISGF3G complex, which then translocates to the nucleus and binds to IFN-responsive elements (ISREs) present in the promoters of over 500 ISGs. Type II IFN binds to the heterodimeric IFNγR1/IFNγR2 receptor also inducing phosphorylation of JAK1/JAK2 kinases. In turn, mostly STAT1 is phosphorylated. Phosphorylated homodimers of STAT1 translocate to the nucleus and induce the expression of genes controlled by gamma-activated sequence (GAS)-dependent promoter sequences. IFNAR1/2, IFN alpha receptor1/2; IFNγR1/2, IFN-gamma receptor1/2; IFNALR1, IFN-lambda receptor 1; IL10R2, IL10 receptor 2; ISGs, IFN-stimulated genes; ISGF3G, ISG factor 3 gamma; JAK1/2, Janus kinase 1/2; STAT, signal transducer and activator of transcription.

Early protection against viral infection is fundamentally mediated by the action of interferons (IFNs), the pillar molecules of the innate immune system (12–14). Expression of IFN is triggered by the recognition of molecular signatures, collectively named pathogen-associated molecular patterns (PAMPs), *via* cellular receptors, pattern recognition receptors (PRRs) that can distinguish “self from non-self” molecules (Figure 1). Binding of PAMPs to PRRs triggers a series of signal transduction events and posttranslational modifications (PTMs: phosphorylation, ubiquitination, ISGylation, etc.) that ultimately activate latent transcription factors to induce IFN transcription. Subsequently, secreted IFN proteins bind to specific receptors on the plasma membrane to activate, in an autocrine and paracrine manner, discrete and overlapping cellular signal transduction pathways. Depending on the cell type and affected tissue, over 500 specific IFN-stimulated genes (ISGs) may be induced, many of which display antiviral activity to control the viral infection (12, 15, 16). There are three families of IFNs based on the specific receptor usage: types I, II, and III (Table 1) (13, 43–50). Type I IFNs (i.e., IFN-α and IFN-β) signal through a heterodimeric receptor complex formed by IFNAR1/IFNAR2, type II IFN (IFN-γ) signals through the complex IFN-γR1/IFN-γR2, and type III IFNs bind the receptor complex IL-28Rα/IL-10Rβ. Despite the receptor differences, the three IFN families transduce signals through the Janus kinase (JAK)–signal transducer and activator of transcription (STAT) pathway, and type I and type III IFNs induce redundant responses (Figure 2). Overall, the rapid production of IFN helps to limit viral replication while modulating other immune functions.

**Table 1:** Use of IFN-based therapies against FMDV.

**Type**	**Recept**.	**Signal**	**Sub-type**	**Species**	**Milestone**
Type I	*IFNAR1/IFNAR2*	*JAK1, TYK2*	*IFN-α/β*	Porcine/bovine	• Recombinant bacterial expressed IFN-α/β is a potent biotherapeutic against FMDV *in vitro* (17)
			*IFN-α*	Porcine	• Ad5 delivered poIFN-α protects swine against different serotypes of FMDV (18–20)
					• poIFN-α-protection correlates with enhanced tissue-specific innate immune cell infiltration in swine (21, 22)
					• poIFN-α protection correlates with upregulation of essential ISGs *in vitro* (23, 24)
			*IFN-β*	Porcine	• Ad5 delivered porcine poIFN-β protects swine against FMDV (20)
			*IFN-δ*	Porcine	• Bacterially expressed poIFN-δ8 significantly inhibits FMDV replication *in vitro* (25)
			*IFN-ω7*	Porcine	• *E*. coli produced poIFN-ω7 protects cells against FMDV (26)
			*IFN-αω*	Porcine	• Bacterially expressed IFN-αω added prior to infection resulted in a significant reduction in FMDV replication *in vitro* (27)
			*IFN-τ*	Ovine	• Ovine IFN-τ has antiviral effect against FMDV *in vitro* (28)
Type II	*IFNγR1 IFNγR2*	*JAK1, JAK2*	*IFN-γ*	Bovine	• Recombinant bovine IFN-γ reduced FMDV replication in BTY cell culture (29)
			*IFN-γ*	Porcine	• High dose of Ad5-poIFN-γ protects swine against FMD (30)
Type III	*IFN-λR1/IL-10R2*	*JAK2, TYK2*	*IFN-λ1*	Porcine	• Replication of FMDV in IBRS-2 cells is inhibited by treatment with the purified recombinant poIFN-λ1 (31)
			*IFN-λ3*	Bovine	• Inoculation with Ad5-boIFN-λ3 resulted in the induction of several ISGs in tissues of the upper respiratory tract (32) and protected cattle against challenge with FMDV (33)
				Porcine	• Ad5-poIFN-λ3 protects swine against challenge with FMDV (34)
IFN Combos			*IFN-α IFN-γ*	Porcine	• Use of a combination of Ad5-poIFN-γ and Ad5-poIFN-α (30) or Ad5-poIFN-αγ (35) showed an enhancement of the antiviral activity against FMDV in swine
Other			*Poly IC*	Porcine	• Double stranded (ds) RNA poly ICLC, in combination with Ad5-poIFN-α protected swine against FMDV (36)
			*siRNA*	Porcine	• Combination of Ad5-poIFN-αγ with Ad-3siRNA targeting FMDV NS coding regions blocked replication of all serotypes of FMDV *in vitro* (37)
			*IRF7/3*	Porcine	• Inoculation with Ad5-IRF7/3(5D) resulted in induction of IFN-α and fully protected mice and swine challenged with FMDV 1 day after treatment (38, 39)
			*IRES*	Porcine	• Use of synthetic IRES in combination with adjuvanted type-O FMD, improved immune response and protection against FMDV challenge (40)
IFN/vaccine combos			*IFN-α*	Porcine	• Use of a combination of Ad5-po-IFN-α and Ad5-A24 in swine resulted in complete protection after challenge (19)
			*IFNα/γ*	Porcine	• Ad5-poIFNα/γ co-administered with Ad5-siRNA targeting NS regions of FMDV, and a commercial inactivated FMD vaccine partially protected swine (41)
			*IFN-λ3*	Bovine	• Use of a combination of Ad5-bov-IFN-γ3 and Adt-O1M in cattle resulted in complete protection after aerosol challenge (42)

## Foot-And-Mouth Disease Virus Impairs Innate Immunity Molecular Interactions

Recognition of FMDV RNA by the host cell results in the establishment of a rapid antiviral state to limit and control infection. This selective pressure has allowed FMDV to evolve many strategies to ensure enhanced virulence and rapid infectivity. In general, RNA viruses can bypass the IFN response by blocking: (i) global cellular transcription and translation; (ii) IFN induction; and (iii) IFN signaling. Similarly to other RNA viruses, FMDV can also target IFN-independent antiviral responses mostly associated with cellular metabolic functions (i.e., autophagy, apoptosis, stress granule formation, etc.) that have been extensively described elsewhere (51, 52). In this section, we will summarize the current literature on studies conducted *in vitro* that explain how FMDV counteracts the host innate immune response at the molecular level, including RNA sensing, activation of adaptor/effector proteins, and regulation of signaling pathways by specific PTMs.

### Block on Cellular Transcription and Translation

FMDV inhibition of cellular gene expression and protein synthesis during infection is mainly driven by the viral-encoded proteases: Leader (Lpro) and 3C. FMDV Lpro is a papain-like protease (PLP) that induces cleavage of the translation initiation factor eIF4G, including eIF4GI and eIF4GII (53, 54) to disable cap-dependent protein synthesis. Also, FMDV Lpro causes degradation of the transcription factor nuclear factor (NF)-κB and results in blockage of specific downstream signaling effectors (55, 56). Studies in porcine cells demonstrated that FMDV Lpro can promote its self-binding to the transcription factor activity-dependent neuroprotective protein (ADNP) and negatively regulate the activity of the IFN-α promoter (57). In contrast, chromatin changes that favor the upregulation of IFN and ISGs can inhibit FMDV replication (58). Interestingly, deletion or mutations in different domains of Lpro result in viral attenuation *in vitro* and *in vivo* (59–63). Furthermore, these studies have shown a strong type I IFN activity upon infection with different versions of FMDV Lpro mutants (23, 56, 61).

Interruption of cellular translation during infection can also be mediated by FMDV 3Cpro, a chymotrypsin-like cysteine protease that similarly to Lpro targets eIF4G and the cap-binding complex eIF4A for cleavage, although these events occur later in the infection (64, 65). 3Cpro can also participate in the inhibition of host–cell transcription by cleaving histone H3 upon FMDV infection (66, 67).

### Block on Interferon Induction

During infection, the initial event that leads to the production of IFN and pro-inflammatory cytokines is the recognition of viral RNA (Figure 1). Sensing of FMDV-RNA is mediated by MDA5 (68), a protein that belongs to a family of helicases known as retinoic acid-inducible gene-I (RIG-I)-like receptors (RLRs). Recent studies have shown that the interaction between RLRs (RIG-I and LGP2) and the FMDV proteins Lpro, 2B, and 3A interferes with the induction of type I IFN (69–72). Indeed, overexpression of either FMDV 2B or 3A resulted in the downregulation of RIG-I and MDA5 mRNA expression (69, 70). In contrast, upregulation of LGP2 transcripts has been observed during FMDV infection in porcine cells, despite a detectable reduction of LGP2 protein levels, presumably due to FMDV Lpro-induced cleavage (71, 72). The apparent inconsistency between the levels of LGP2 mRNA and protein during FMDV infection may be explained by LGP2's ability to serve as a positive and negative regulator of RIG-I and MDA5 signaling, presumably affecting multiple steps of the IFN induction pathway (73). In addition to RLRs, nucleotide-binding oligomerization domain (NOD)-like receptors (NLRs), NOD1 and NOD2, also participate in the recognition of RNA. A study by Liu et al. (74) described the association of NOD2 with FMDV 2B, 2C, and 3Cpro to block innate immunity activation. Protein kinase R (PKR) is another recognized PRR that acts as an RNA sensor (75). Binding of RNA to PKR induces a conformational change that leads to autophosphorylation and activation (76). The primary target of activated PKR is the eukaryotic initiation factor 2 α subunit (eIF2α), whose phosphorylation results in the blockage of cellular protein synthesis, a relatively common process during viral infection (77). Although no direct interaction between FMDV RNA and PKR has been demonstrated, it has been reported that PKR activity modulates FMDV infectivity. In fact, in tissue culture experiments, depletion of endogenous levels of PKR using siRNA resulted in increased FMDV titers (17, 23). Furthermore, it has been recently shown that overexpression of autophagy-related ATG5-ATG12 proteins induces transcription of PKR and subsequent reduction of FMDV replication (78). These results suggest that PKR has a complex role as an RNA sensor but also as an antiviral agent during FMDV infection.

It has been demonstrated that FMDV also targets DExD/H-box RNA helicases, formally accepted as PRRs and modulators of the antiviral signaling pathway (79). *In vitro* experiments intending to analyze protein–protein interactions revealed the association between the RNA helicase DDX1 and FMDV 3D (80). Interestingly, these studies indicated that during FMDV infection in porcine cells, cleavage of DDX1 was detected, while overexpression of DDX1 resulted in the upregulation of IFN-β and other ISG mRNAs which correlated with virus inhibition (80). Other DExD/H-box RNA helicases such as RNA helicase H (RHA) are hijacked during FMDV infection and interact with FMDV 5'UTR, 2C, and 3A to facilitate virus replication (81).

Signaling pathways downstream from RNA sensing involve the activation of different adaptor and effector proteins. One of the pathways that lead to signal activation requires the formation of specific complexes such as NF-κB essential modulator (NEMO) and the kinase IKK, which bridges the activation of NF-κB and IFN regulatory factor (IRF) signaling pathways. It has been demonstrated that FMDV 3Cpro interacts with NEMO and induces its cleavage, resulting in impaired innate immune signaling (82). IRF-mediated signals driven by IRF-3 and IRF-7 can also be targeted by FMDV proteins. Specifically, overexpression of Lpro in PK-15 cells resulted in the downregulation of IRF-3 and IRF-7 protein levels and inactivation of IFN-β and IFN-λ1 promoter (31, 83).

Other factors involved in the activation of IFN include conventional PTMs such as phosphorylation and ubiquitination which ensure effective regulation of these signaling pathways (84). Also, different cellular deubiquitinases (DUBs) can reverse ubiquitination to control the intensity of the immune signaling response. Interestingly, it has been shown that FMDV Lpro can remove ubiquitin (Ub) molecules from several proteins required for IFN mRNA expression and those involved in the activation/repression of the IFN loop (85). This role became more evident by the observation that during infection, FMDV Lpro can cleave cellular substrates modified with the Ub-like molecule ISG15 (86). Furthermore, mutation of Lpro that impairs deISGylase/DUB function results in viral attenuation (87). In this regard, identification of FMDV targets for deubiquitination and deISGylation may contribute to elucidate the role of those factors in counteracting the innate response and develop novel countermeasures.

### Block on Interferon Signaling

The ligand-mediated association of the specific IFN receptors promotes a signaling cascade that results in the phosphorylation of the receptor by the action of JAKs. These events result in the generation of docking sites for downstream adaptor and effector proteins including signal transducer and activator of transcription (STAT) proteins that associate with other factors and translocate to the nucleus inducing transcription of a plethora of ISGs (described above and in Figure 2). Although blockage of the JAK–STAT signaling pathway has not been reported during FMDV infection, overexpression of either FMDV 3Cpro or VP3 can inhibit this response. For instance, IFN-β-treated HeLa cells overexpressing FMDV 3Cpro suppressed IFN-stimulated promoter activities and induced proteasome- and caspase-independent protein degradation of karyopherin α1 (KPNA1), the nuclear localization signal receptor for tyrosine-phosphorylated STAT1 (88). This interaction inhibited the nuclear translocation of STAT1/STAT2, impeding maximal ISG promoter activity. In another study in HEK293T cells, overexpression of VP3 followed by co-immunoprecipitation revealed the association between VP3 and JAK1. FMDV VP3 also inhibited virus-triggered activation of the IFN-β promoter, leading to the decrease in transcription of ISGs presumably due to lysosomal-induced degradation of JAK1 (89). A yeast two-hybrid screen identified FMDV 2C in complex with N-myc and STAT interactor (Nmi), a protein known to augment immune function dependent on STAT-mediated transcription. Interestingly, such interaction resulted in the recruitment of Nmi to vesicular compartments followed by the induction of apoptosis in BHK-21 cells (90).

Evidently, FMDV proteins can also target crosstalk pathways induced by JAK/STAT signaling, and due to this versatility, understanding of these signaling events during FMDV infection is challenging.

## Foot-And-Mouth Disease Virus Impairs Interferon-Mediated Cellular Innate Immune Responses

Similarly to what happens *in vitro*, FMDV manipulates the early innate immune response *in vivo* to ensure a window of opportunity that favors viral replication and spread before the onset of effective adaptive immunity required for virus clearance. During infection, FMDV interacts with a range of host cells including natural killer (NK) cells, dendritic cells (DCs), monocytes/Mϕ, and γδ T cells. All these cells play an important role in innate immune responses that trigger the production of large quantities of IFN and other cytokines which serve as autocrine agents (91–95).

Shortly after FMDV infection in swine, the number of circulating NK cells transiently decreases and the remaining NK cells show a dysfunctional lytic activity against target cells and a reduction of IFN-γ production (96). In parallel, FMDV blocks the ability of porcine DCs to mature into conventional DCs (cDCs) (97), dampening their response against Toll-like receptor (TLR) ligands (98). Another subset of porcine DCs, plasmacytoid DCs (pDCs), also referred to as the major professional systemic IFN-α producers, are also affected by FMDV (99, 100). During infection, partial depletion of pDCs in the peripheral blood has been detected, and the remaining pDCs are less capable of producing IFN-α in response to *ex vivo* stimulation by TLR ligands or virus (101). Similar to pDCs, FMDV infection reduces the production of IFN-α on Langerhans cells (LCs) (98), a distinct subset of tissue-resident DCs of the skin (102). It has also been suggested that porcine γδ T cells and Mϕ can serve as targets for FMDV infection in swine (103, 104), although the interplay between these cells and FMDV remains unclear.

Comparably to swine, FMDV infection in cattle triggers several early events in the innate immune system, although the effects are not exactly the same. For instance, bovine NK cells originated from FMDV-infected cows have an elevated cytotoxic function against bovine target cells *in vitro* (105). In addition, some subsets of cDCs are significantly decreased during the peak of viremia, while the expression of major histocompatibility complex (MHC) class II molecules on all bovine cDCs is reduced and the processing of exogenous antigen is impaired (106). Furthermore, during FMDV infection, the number of systemic mature bovine pDCs characterized by the expression of CD4+ and MHC class II+ is increased presumably to intensify a humoral response and T cell activation, while levels of immature CD4+ MHC class II-pDCs are declined (106). Examination of bovine γδ T cells revealed that these cells with the surface expression marker WC1+ show a transient activated phenotype and increased expression of IFN-γ (107).

FMDV also affects the innate immune response at the cytokine level in the natural host. *In vivo* cytokine profile analysis during the clinical phase of disease shows a systemic decrease of pro-inflammatory cytokines [IL-1β, IL-6, and tumor necrosis factor (TNF)-α] and an increase of the anti-inflammatory cytokine IL-10 and IFN-α (22, 33, 61, 101, 106). Most likely, these changes are related to the early T cell unresponsiveness and lymphopenia described in swine and cattle during FMDV infection (33, 102, 106, 108). Interestingly, a significant induction of inflammatory and antiviral factors at the local level is detected in cattle, in sites of abundant viral amplification, such as the nasal/oropharynx or vesicular lesions (109–111). A consistent upregulation of IFN-α, -β, -γ, and -λ mRNA in distinct microanatomical compartments of the nasopharyngeal mucosa, concurrent with occurrence of viremia, has also been detected in cattle (112). In contrast, studies in swine demonstrated that IFN expression in infected swine skin is inhibited (21). These differences may be due to the analysis of follicle-associated epithelium of the nasopharyngeal mucosa in cattle vs skin in swine or to the specific sampling technique used in each experiment. While in the cattle study laser-capture microscopy was used to focus only in areas of high FMDV replication, in the swine study, RNA was extracted from a piece of skin without discriminating between microanatomical compartments. Evidently, more studies are needed to elucidate the intricate interactions between FMDV and the innate immune system of specific animal hosts.

## Effective Use of Interferon Against Foot-And-Mouth Disease Virus *In vitro*

### Type I Interferon

The role of IFN in controlling FMDV replication was first proposed in 1962 when Dinter and Philipson demonstrated that calf kidney cells exposed to FMDV could become persistently infected and proposed this was a consequence of the induction of an IFN-like inhibitor present in the supernatant of infected cells (113). Later studies also suggested that swine leukocytes treated with phytohemagglutinin produced an inhibitor of FMDV replication with properties similar to IFN (114). It was not until 1999 that new studies demonstrated that the ability of FMDV to form plaques in cell culture correlated with the suppression of type I IFN (α/β) protein expression (115). These results were further supported by detection of IFN protein and antiviral activity in the supernatants of primary porcine, ovine, and bovine kidney cells infected with an attenuated FMDV mutant (leaderless) as compared to the supernatants of cells infected with wild-type (WT) virus. Later studies by the same group provided proof of concept on the use of recombinant bacterial expressed IFN-α/β as a potent biotherapeutic against FMDV (17). This approach was further developed by delivering recombinant porcine IFN-α/β using a replication-defective human Adenovirus 5 vector (Ad5-poIFN-α/β) (18). Infection of IBRS-2 cells with Ad5-poIFN-α/β resulted in secreted poIFN-α/β IFN protein detected as early as 4 h post-infection (hpi) and lasting for at least 30 h. Most important, expressed IFN protein displayed strong biological antiviral activity against FMDV. Follow-up studies by the same group showed that all FMDV serotypes are very sensitive to Ad5-delivered poIFN-α/β, and sterile protection could be achieved *in vivo*, highlighting the potential of this approach for the development into a broad biotherapeutic strategy to control FMDV replication (116).

In the last 10 years, advancements is genomics have led to the characterization of almost all type I IFN subtypes in the porcine and bovine genome (117–119), which are more numerous than those identified in primates and mice. This has revealed different functional genes and pseudogenes with diverse expression profiles and antiviral functions against different viruses, mostly in swine (118, 120, 121). In fact, a recent study demonstrated that poIFN-ω7, known for its ability to induce the highest levels of antiviral activity when compared to other poIFN-ω subtypes, elicits an antiviral state against FMDV in IBRS-2 cells treated with the recombinant form of poIFN-ω7 produced in *Escherichia coli* (26). Other subclasses of type I IFN, known to be produced in swine and cattle, include IFN alphaomega (IFN-αω, also known as IFN-μ) and IFN delta (IFN-δ). Significant reduction in FMDV replication has been observed upon treatment of porcine cells with bacterially expressed IFN-αω or IFN-δ8 prior to viral infection (25, 27).

Recently, another member of type I IFN family, IFN-τ, which is only produced in ruminants, has been evaluated as an antiviral against FMDV (28). IFN-τ is a paracrine reproductive hormone secreted constitutively by trophoblasts and endometrial cells to increase the life span of the corpus luteum; however, production is not induced upon viral infection (122). While its secretion is restricted to ruminants, it has a broad-spectrum activity against various cross-species viruses. Interestingly, IFN-τ has 55% homology with the amino acids of IFN-α, which allows for binding to type I IFN receptors. The property of IFN-τ that makes it an interesting therapeutic candidate for the treatment of various viral diseases is its significantly lower toxicity as compared to other type I IFNs.

### Type II Interferon

In contrast to type I IFN, the type II IFN family is composed of only one member, IFN-γ, which exerts its actions through a specific receptor, IFNGR1/IFNGR2. IFN-γ is weakly resistant to heat and acid, and it is able to activate leukocytes such as macrophages, and granulocytes, also exerting regulatory functions on T and B lymphocytes (123, 124). Indeed, production of IFN-γ is used as a tool to measure cell-mediated immune responses against FMDV in vaccinated cattle (125–127) and in swine (61). Interestingly, IFN-γ responses as measured by its ability to induce proliferation of CD4+ T cells correlate with a vaccine-induced protection and a reduction of FMDV persistence as it was shown for bovines inoculated with high doses of inactivated vaccine FMDV A Malaysia 97 (128). Therefore, the increase of the cellular immune response against FMDV seems to be comparable with the upregulation of IFN-γ at least in cattle (125, 127, 128).

One of the first experiments that examined the IFN-γ potential to inhibit FMDV replication was performed in bovine thyroid (BTY) cells. BTY cells were treated with different concentrations of recombinant bovine IFN-γ followed by infection with FMDV variants isolated from oropharynx cells collected from persistently infected bovines (29). Interestingly, IFN-γ pretreatment resulted in a significant reduction of viral RNA and FMDV proteins as measured by RT-PCR and ELISA, respectively. These results were further bolstered by experiments intended to provide insights on the molecular mechanism of the IFN-γ antiviral function against FMDV. Specifically, a transcriptomic analysis of FMDV-infected porcine kidney cells previously treated with IFN-γ revealed a significant upregulation of transcription factors (STAT1 and IRF1) involved in the regulation of diverse ISGs (129). By using the Ad5 vector strategy, it was also demonstrated that type II IFN displays antiviral activity against FMDV in porcine cells (19). Interestingly, significant enhancement of the antiviral effect against FMDV was observed by using a combination of Ad5-poIFN-γ and Ad5-poIFN-α. Furthermore, use of a dicistronic Ad5 vector that expresses both poIFN-γ and poIFN-α has shown enhanced antiviral activity in porcine cells (35).

### Type III Interferon

The newest addition to the IFN families is the type III IFNs (IFN-λ1 or IL29, IFN-λ2 or IL28A, IFN-λ3 or IL28B, and IFN-λ4) which share signal transduction pathways of the type I IFN family albeit the use of a different cellular receptor, the IL-28Rα/IL-10Rβ heterodimer. In contrast to type I IFN receptors, which are expressed in almost all cell types, IL-28Rα is expressed in a tissue-dependent fashion such as epithelia (49, 50). In addition, downstream activation of IFN-λ-induced signals requires phosphorylation of STAT1 mediated by JAK2 (130). The first study that reported the antiviral function of IFN-λ against FMDV was conducted in bovine cell cultures (32). In this study, embryonic bovine kidney (EBK) cells treated with supernatants from cells previously transduced with Ad5-boIFN-λ3 protected cells from FMDV-induced cytopathic effects and correlated with enhanced upregulation of IFN and ISG mRNAs. Similarly, porcine cells could be protected against FMDV infection by pretreatment with recombinant porcine IFN-λ1 (poIFN-λ1) (31) or with supernatants of cells transduced with an Ad5-poIFN-λ3 (34). All together, these results demonstrated that FMDV is highly susceptible to the action of type III IFN.

## Effective Use of Interferon Against Foot-And-Mouth Disease Virus in the Natural Host

Despite distinct induction of IFN and innate immune responses during FMDV infection in swine and cattle, spatial distribution of IFN is similar. In both species, *in vivo* detection of IFN occurs only after the virus has successfully replicated in the primary site and has spread systemically. In fact, similarly to what has been described *in vitro* (see previous section), the virus is very sensitive to the IFN antiviral effect *in vivo* (22). This property highlighted the potential use of these molecules as biotherapeutics against FMD, inviting new research to evaluate similar products against emerging animal diseases, a policy supported by the OIE. However, the use of IFNs in animals requires extensive testing in species of interest in order to evaluate the metabolic rate and potential adverse systemic effects of individual preparations (131–133). In this regard, although only in humans or animal models for human diseases, many approaches to change IFN's pharmacokinetic profile have been examined. These include the covalent modification of IFN with poly-ethylene-glycol (PEG) molecules (PEGylation) or the expression of recombinant IFN fused to Fc fragments of immunoglobulins. Evaluations of these modified IFNs have been tested for the treatment of multiple human diseases such as hepatitis B and C, multiple sclerosis, and cancer (134–137). Potential use of these new IFN-modified platforms should improve its biotherapeutic function in the animal setting.

In this section, we summarize *in vivo* studies that evaluated the use of different platforms to deliver IFN or IFN inducers, alone or in combination, as a means to protect against FMD (Table 1).

### Interferon Treatment Protects Swine Against Foot-And-Mouth Disease

The first IFN tested in swine for its antiviral activity against FMDV was poIFN-α, delivered with an Ad5 vector (18). Using this platform, swine intramuscularly (IM) inoculated with 10^9^ pfu of Ad5-poIFN-α expressed relatively high levels of systemic antiviral activity detectable as early as 6 hpi and lasting for 72 h. These results correlated with complete protection against intradermal (ID) challenge with FMDV A24 at 24 h post Ad5-poIFN-α inoculation (18). Furthermore, complete protection lasted for 3–5 days, causing a delay in disease onset, reduced severity of clinical signs, and a significant reduction in viremia even when FMDV challenge was performed at 7 days post inoculation (dpi) or 1 day prior to the treatment (19). Extensive studies in swine using this vector or a modified proprietary version of Ad5 (Adt-poIFN, GenVec®) demonstrated that delivery of poIFN-β was also effective against challenge with FMDV at 1 dpi. Remarkably, depending on the administered Adt-poIFN dose, treated animals could be sterilely protected against FMD based on standardized parameters (20).

One of the advantages of using IFN against FMDV is the high likelihood for viral clearance regardless of the specific serotype (1, 138). In fact, swine experiments in which animals were inoculated with Ad5-pIFN-α and challenged intradermally (ID) 24 h later with different FMDV serotypes, A24, O1 Manisa or Asia, showed the same level of protection (20). Importantly, when the challenge was performed using a contact challenge, a route of inoculation that resembles the natural FMDV infection in swine (139–141), similar results were obtained (20).

Studies to understand the mechanisms of protection induced by type I IFN in swine demonstrated that protection of swine inoculated with Ad5-poIFN-α correlated with recruitment of partially mature skin DCs showing increased expression of CD80/86 and decreased phagocytic activity (21, 22). At the same time, an increase in the number of NK cells in draining lymph nodes was noticeable (21). These findings corresponded with upregulation of a number of ISGs, including PKR and 2′-5′-oligoadenylate synthase (OAS), which block FMDV replication in cell culture (17, 23). Other cytokines and chemokines, including monocyte chemotactic protein-1 (MCP-1), macrophage inflammatory protein (MIP)-1α, and IFN inducible protein 10 (IP-10) which are involved in chemoattraction of DCs and NK cells (142), were also upregulated. Interestingly, using a mouse model for FMDV (143), it was shown that IP-10 is necessary for protection conferred by murine IFN-α (muIFN-α), since C57Bl/6-IP-10 knockout mice treated with muIFN-α prior to challenge were not protected against disease, whereas C57Bl/6-WT mice pretreated in the same way, were completely protected (24).

The effect of type II IFN has also been tested in swine using the Ad5 platform for delivery of IFN-γ (30). Animals IM inoculated with 10^10^ pfu of Ad5-poIFN-γ were protected against challenge at 1 dpi. Interestingly, enhanced antiviral activity was observed when a combination of Ad5-poIFN-α and Ad5-poIFN-γ was administered, allowing for Ad5-IFN vector dose sparing to fully protect swine against challenge with FMDV A24 at 1 dpi (30). More recently, Kim et al. (35) used a similar approach against FMDV O1 in swine. Enhancement of potency against FMD was observed upon treatment with an Ad5 vector that expressed bicistronically poIFN-α and IFN-γ, as compared to either IFN alone (35).

The type III family of IFNs also has an antiviral effect against FMDV *in vivo*. Swine inoculated with Ad5-poIFN-λ3 and exposed 1 day later to FMDV by contact exposure to infected swine were completely protected from clinical disease, with no detectable viremia, viral RNA, or virus shedding (34). Interestingly, protection was achieved even when systemic antiviral activity or upregulation of ISGs in peripheral blood mononuclear cells (PBMCs) were undetected. This was consistent with previous reports indicating that expression of the IFN-λ receptors (IFN-λR1) and sensitivity to IFN-λ are highest in epithelial tissues and not in leukocytes (144, 145).

Additional IFN-based therapeutics have been used *in vivo* in swine. These strategies were directed toward the use of synthetic nucleic acids that would mimic viral PAMPs or could interfere with the expression of specific viral genes without triggering the IFN response. In addition, construction of Ad5 vectors that deliver transcription factors or other antiviral factors involved in the production of IFN has been tested.

Use of nucleic acid-based molecules including the synthetic double-stranded polyriboinosinic-polyribocytidylic acid molecule stabilized with poly-L-lysine and carboxymethylcellulose (polyICLC) in combination with Ad5-poIFN-α protected swine against FMDV challenge as these animals developed the highest levels of antiviral activity along with detectable poIFN-α in the blood (36). This is in contrast with original studies done in pigs where intravenous inoculation of polyIC alone did not result in protection (146), highlighting the importance of the route of administration and immunity. Other studies have demonstrated that inoculation of mice with *in vitro*-transcribed RNAs mimicking some structural domains contained within the 5' and 3' non-coding FMDV UTRs can induce stable and robust production of systemic type I IFN (147). Moreover, the same group showed that delivery of a synthetic RNA, corresponding to 470 nt of the FMDV internal ribosome entry site (IRES), improves the immune response induced in mice in terms of timing, magnitude, and endurance of specific antibody titers (148). More recently, the same approach was evaluated in swine. Inoculation with the synthetic IRES transcript in combination with an adjuvanted type-O FMD vaccine resulted in an improved immune response and protection against FMDV challenge as compared to inoculation with the same vaccine alone (40). Interestingly, administration of this vaccine combination resulted in enhanced specific B and T cell-mediated immune responses as compared to suboptimal doses of the vaccine alone (40).

Additionally, Ad5 delivery of small interfering RNAs (siRNAs) targeting FMDV structural and NS coding regions protected swine against FMDV (149), even when animals were treated 3 days after the challenge (150).

Due to the high mutation rate inherent to RNA viruses (151, 152), use of antivirals can result in virus adaptability. Studies by Kim et al. (35, 37, 153) have proposed that the combination of antivirals including siRNA, viral polymerase inhibitors (i.e., ribavirin), and IFNs is better suited to minimize the generation of FMDV-resistant mutants. For instance, combination of Ad5-poIFN-α/γ with an Ad5 expressing three different siRNAs (Ad5-3siRNA) targeting FMDV NS coding regions (2B and 3C) was effective against all serotypes of FMDV in swine cells (37). Thus, a combined treatment with Ad5-poIFN-α/γ and Ad5-3siRNA could work as a fast-acting antiviral treatment to induce protection prior to the induction of vaccine-mediated adaptive immunity.

Another approach known to induce an early broad innate immune response is the use of replicon vaccine vector systems, such as the Venezuelan equine encephalitis virus (VEE) replicon particles (VRPs) (154). Treatment with this biotherapeutic platform results in the upregulation of a number of ISGs and the production of type I IFN protein (155) and has been tested successfully against FMDV *in vitro* and *in vivo* using a mouse model (24).

Baculovirus-based strategies have also proved successful in mice against FMD based on their robust IFN induction capacity. Molinari et al. (156) demonstrated that pretreatment of C57Bl/6 mice with a single injection of *Autographa californica* nuclear polyhedrosis virus (AcNPV) at 3 h or 3 days before FMDV challenge prevented animal death and decreased symptoms of disease and viremia. Further, treatment of mice with a combination of AcNPV and vaccine conferred early and full protection against lethal FMDV challenge (157).

More recently, a constitutively active transcription factor, IRF7/3(5D) fusion protein was explored as a means to induce innate responses against FMDV. *In vivo* delivery of IRF7/3 (5D) using the Ad5 vectored expression system resulted in potent induction of IFN-α and complete protection against FMDV in mice and swine (38, 39).

### Interferon Treatment Protects Cattle Against Foot-And-Mouth Disease

Although the use of type I IFN using the Ad5 platform has been proven very successful in swine, preventive therapy only had limited efficacy in cattle. Inoculation of bovines with high doses of Ad5-poIFN-α or Ad5-bovine IFN-α (Ad5-boIFN-α) induced a relatively low level of systemic antiviral activity (100–200 U/ml), and challenge of these animals with FMDV A24 by intradermolingual (IDL) inoculation only resulted in a short delay and reduced severity of disease as compared to control animals (158).

In contrast, in preliminary experiments, the use of the type III IFN in bovine proved to be more successful than the use of type I IFN (32), although inoculation of cattle with Ad5-boIFN-λ3 resulted in low levels of systemic antiviral activity. Interestingly, induction of several ISGs was detected in tissues of the upper respiratory tract, known targets of FMDV. An enhanced effect in ISG upregulation was detected when animals were treated with a combination of Ad5 vectors expressing type I and III IFNs. Inoculation of cattle with high doses of Ad5-boIFN-λ3 followed by FMDV IDL challenge at 24 hpi resulted in a significant delay (6–12 days) and reduced severity of disease (33). Furthermore, a stronger effect was detected when treated cattle were challenged by aerosolization of FMDV using a method that best resembles the natural route of infection (140). No clinical signs of FMD, viremia, or viral shedding were found in the Ad5-boIFN-λ3-treated animals for at least 9 days post-challenge, and one of three inoculated animals remained free of disease during the entire experiment (33). These results indicated that boIFN-λ3 plays a critical role in the innate immune response of cattle against FMDV, and treatment with Ad5-boIFN-λ3 is an effective biotherapeutic approach to control FMD in bovines.

## Combination of Interferon and Foot-And-Mouth Disease Vaccine as an Approach to Fully Protect Livestock Against Foot-And-Mouth Disease Virus

A complete control strategy would ideally include both, a rapid-acting approach to immediately limit disease spread, and a long-lasting preventive measure to protect livestock from further exposure to FMDV. Therefore, it is reasonable to consider that a combination treatment of IFN and vaccine would be the best strategy to control FMD. In proof-of-concept studies in swine, a combination of Ad5-poIFN-α and an Ad5 vaccine that delivers structural and capsid processing proteins of FMDV A24 (Ad5-FMD-A24) resulted in complete protection when animals were challenged at 1–5 dpi while a strong adaptive immune response was induced (19). Using a comparable platform, a combination of Ad5-boIFN-λ3 and Ad5-FMD-O1M had a similar performance in cattle. In this experiment, complete protection was achieved after animals were exposed to FMDV by aerosol (42). Remarkably, protection of animals treated with the combination occurred despite the absence of detectable neutralizing antibodies or antiviral activity in serum at the time of the challenge (42). Although not proved in this study, it is possible that the remaining antiviral activity at the mucosal level was able to block FMDV replication, as described for type III IFN during rotavirus infection (159). However, exploring other protective mechanisms such as cellular immunity should also be considered to understand this protection. Other strategies that have been explored *in vivo* include the simultaneous treatment with an Ad5 that delivers poIFN-α and FMDV VP1, but this study was performed in mice and it was not followed up with experiments in the natural FMDV host (160). More recently, You et al. (41) tested in swine the efficacy of the combined treatment with three antivirals, Ad5-poIFNα/γ co-administered with Ad5-siRNA, and a commercial inactivated FMD vaccine, however, only partial protection was observed when challenge was performed at 1, 2, or 7 days post-vaccination (dpv) (41). All together, these results indicate that a combination treatment of IFN and vaccine is a desirable strategy that could be used to fully protect cattle and swine from FMD.

## Concluding Remarks and Future Perspectives

Over the past 20 years, considerable progress has been made in the development of IFN-based biotherapeutics to control FMD. The use of different delivery technologies, such as the Ad5 vector, highlighted the ability of IFN to confer protective immunity against FMDV in swine and cattle. Importantly, the identification of different cellular factors and cellular immune responses that are targeted during FMDV infection and affect the IFN system furnished our knowledge of FMDV virulence and pathogenesis. These discoveries permitted the development of new intervention strategies to improve IFN-based therapies such as proper selection of IFN type, evaluation of the route and site of inoculation, and utilization of synthetic IFN inducers that could act as potential adjuvants, augmenting the intrinsic biotherapeutic effect, and also improving FMD vaccine performance. Such strategies seem ideal for application in endemic regions to potentially reduce the number of exposed or at high risk of exposure animals. On the other hand, a similar strategy could be applied in the unfortunate event of outbreaks in FMD-free countries that opt for a vaccination-to-kill policy. In this case, by using an antiviral/vaccine combination approach, disease spread would be more limited, hopefully reducing the economic burden.

However, before IFNs could be used as a gold standard therapeutic agent against FMD, several considerations must be taken. For instance, metabolic rate of absorption and toxicity should be carefully evaluated to finely tune therapeutic doses for each animal species of interest. Study of specific IFN expression profiles and intrinsic antiviral activities in different tissues may also help to improve and optimize treatments for specific animal hosts.

Some of these shortcomings could be aided by selecting the right type and subtype of IFN, depending of the specific animal species of interest. In addition, novel advancements in protein engineering have demonstrated that IFN potency and bioavailability could be improved. In this regard, chemically modified IFN molecules (i.e., PEGylation) or other protein fusions deserve being evaluated as possible interventions for animal diseases. Finally, continuing studies to better characterize innate immune responses during FMDV infection *in vitro* and *in vivo* will help refine our understanding of the anti-FMDV properties of IFN and hopefully develop improved therapeutics for effective FMD control and disease eradication.

## Author Contributions

All authors contributed equally with literature searches, writing, editing, and approving the submission.

## Conflict of Interest

The authors declare that the research was conducted in the absence of any commercial or financial relationships that could be construed as a potential conflict of interest.
